# Influence of Tweets Indicating False Rumors on COVID-19 Vaccination: Case Study

**DOI:** 10.2196/45867

**Published:** 2023-09-05

**Authors:** Mai Hirabayashi, Daisaku Shibata, Emiko Shinohara, Yoshimasa Kawazoe

**Affiliations:** 1 Artificial Intelligence in Healthcare Graduate School of Medicine The University of Tokyo Tokyo Japan

**Keywords:** coronavirus, correlation, COVID-19, disinformation, false information, infodemiology, misinformation, rumor, rumor-indication, SARS-CoV-2, social media, tweet, Twitter, vaccination, vaccine

## Abstract

**Background:**

As of December 2022, the outbreak of COVID-19 showed no sign of abating, continuing to impact people’s lives, livelihoods, economies, and more. Vaccination is an effective way to achieve mass immunity. However, in places such as Japan, where vaccination is voluntary, there are people who choose not to receive the vaccine, even if an effective vaccine is offered. To promote vaccination, it is necessary to clarify what kind of information on social media can influence attitudes toward vaccines.

**Objective:**

False rumors and counterrumors are often posted and spread in large numbers on social media, especially during emergencies. In this paper, we regard tweets that contain questions or point out errors in information as counterrumors. We analyze counterrumors tweets related to the COVID-19 vaccine on Twitter. We aimed to answer the following questions: (1) what kinds of COVID-19 vaccine–related counterrumors were posted on Twitter, and (2) are the posted counterrumors related to social conditions such as vaccination status?

**Methods:**

We use the following data sets: (1) counterrumors automatically collected by the “rumor cloud” (18,593 tweets); and (2) the number of COVID-19 vaccine inoculators from September 27, 2021, to August 15, 2022, published on the Prime Minister’s Office’s website. First, we classified the contents contained in counterrumors. Second, we counted the number of COVID-19 vaccine–related counterrumors from data set 1. Then, we examined the cross-correlation coefficients between the numbers of data sets 1 and 2. Through this verification, we examined the correlation coefficients for the following three periods: (1) the same period of data; (2) the case where the occurrence of the suggestion of counterrumors precedes the vaccination (negative time lag); and (3) the case where the vaccination precedes the occurrence of counterrumors (positive time lag). The data period used for the validation was from October 4, 2021, to April 18, 2022.

**Results:**

Our classification results showed that most counterrumors about the COVID-19 vaccine were negative. Moreover, the correlation coefficients between the number of counterrumors and vaccine inoculators showed significant and strong positive correlations. The correlation coefficient was over 0.7 at −8, −7, and −1 weeks of lag. Results suggest that the number of vaccine inoculators tended to increase with an increase in the number of counterrumors. Significant correlation coefficients of 0.5 to 0.6 were observed for lags of 1 week or more and 2 weeks or more. This implies that an increase in vaccine inoculators increases the number of counterrumors. These results suggest that the increase in the number of counterrumors may have been a factor in inducing vaccination behavior.

**Conclusions:**

Using quantitative data, we were able to reveal how counterrumors influence the vaccination status of the COVID-19 vaccine. We think that our findings would be a foundation for considering countermeasures of vaccination.

## Introduction

Social media platforms such as Twitter and Facebook have become widespread and important tools for information dissemination and retrieval, facilitating real-time communication on the internet [1]. Twitter, especially, has made remarkable progress, enabling users to share information easily, widely, and simultaneously among numerous people. During and immediately after the Great East Japan earthquake on March 11, 2011, using telephones (cellular telephones) and email was difficult due to network congestion, but Twitter was still used on cell phones. People confirmed their safety and requested support from others using the platform. Twitter has become an important communications tool during disaster situations, with various studies analyzing the use of this microblogging site for disasters and infectious disease outbreaks [2-7].

Nevertheless, problems with social media exist, with, for example, some users leaking classified information and spreading false rumors. Studies have examined such rumors, providing various definitions of the term [8-10]. Herein, we define a rumor as “unfounded information” and are unconcerned about the course of its development.

In emergency situations, it is easy for rumors to be generated and transmitted [8], especially on social media. Previous studies have analyzed false rumors in emergency situations or tried to develop systems that detect them automatically [11-13]. In Japan, during actual emergencies such as the Great East Japan Earthquake in March 2011 and the novel coronavirus infection (hereafter referred to as COVID-19), rumors spread through social media resulted in various problems; false rumors were associated with health hazards and panic buying of food and daily commodities. To prevent the impact of such rumors on society as a whole and on individuals, developing measures to counteract rumors on social media is essential.

Even in December 2022, the COVID-19 pandemic showed no signs of abating, and its impact on society continues. With the continuation of the pandemic, various changes have occurred, including the emergence of mutant strains, progress in vaccine development, and the start of vaccination. Vaccination is 1 of the most important factors affecting changes in the situation of infection. Therefore, it is important to analyze the factors that may influence vaccination behavior. Even if vaccine development progresses, herd immunity cannot be gained unless vaccination coverage exceeds a certain threshold. Accordingly, it is important to increase vaccination coverage. One obstacle to increased vaccination coverage is “vaccine hesitancy” [14,15]. The Strategic Advisory Group of Experts on Immunization (SAGE) Working Group on Vaccine Hesitancy, established by the World Health Organization (WHO) within the SAGE in 2012, defines vaccine hesitancy as a “delay in acceptance or refusal of vaccination despite the availability of vaccination services” [16]. Vaccine hesitancy is impacted by various factors, such as sociodemographic, psychosocial, and one’s environment. According to the COVID-19 survey on vaccination intention, the decision to vaccinate is influenced primarily by 2 factors: “the risk of not vaccinating” and “the risk of vaccinating.” The higher the risk of not vaccinating, the more likely a person is to vaccinate [17]. Additionally, the risk perception of side effects may increase for pandemic vaccines that have not been used before because the risk perception increases as uncertainty increases. Other studies indicate that vaccination decisions are influenced by the use of social media and the internet as main sources of information [18].

Uncertain information exists on social media platforms such as Twitter largely because individuals can easily post and spread information. Twitter users also post tweets that contain phrases with questions or errors in information. In this paper, we regard a tweet indicating a false rumor that contains phrases about questions or indications of errors in information as a counterrumor. Tweets containing false rumors and counterrumors about the COVID-19 vaccine may be posted and spread on Twitter. As previously stated, the results of a survey on vaccine hesitancy suggest that the use of social media and the internet are primary sources of information and are factors associated with vaccine hesitancy [18]. Browsing through information on social media has been shown to lead to vaccine hesitancy in Canada [19]. Following these results, we predicted that false rumors and counterrumors about the COVID-19 vaccine on Twitter may influence people’s vaccination behavior in Japan. Due to the COVID-19 pandemic, various studies have analyzed tweets about COVID-19 posted on Twitter [20-26]. These studies focused on analyzing the topics and sentiment of the tweets and did not discuss the effects of social media on vaccinations using quantitative data. In this study, we analyze the characteristics of counterrumors related to the COVID-19 vaccine and discuss whether there is a relation between counterrumors and COVID-19 vaccination behavior. Specifically, we investigate the following two questions: (1) Do counterrumors about the COVID-19 vaccine influence vaccination status? (2) If so, what kind of influence do they have?

## Methods

### Overview

**Figure 1:**
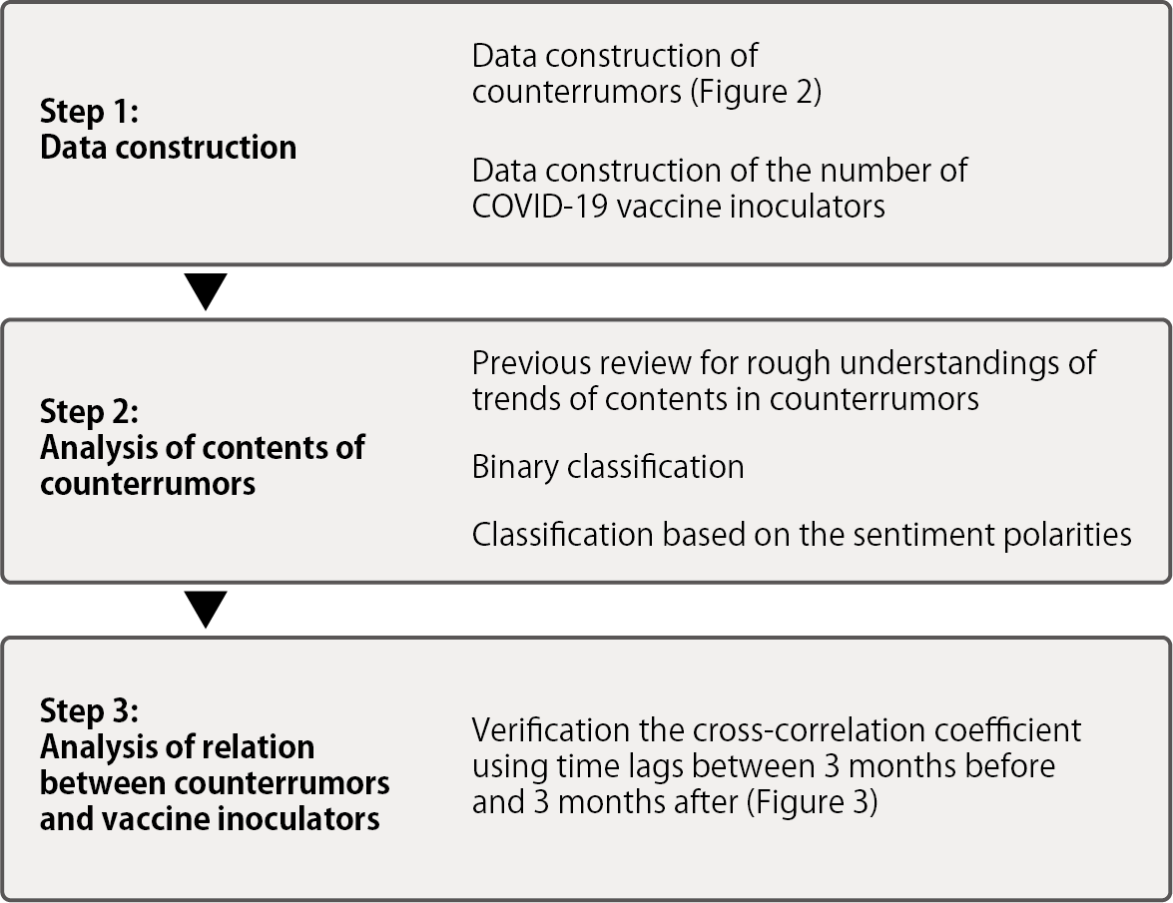
Stepwise approach taken for our work.

Figure 1 shows the stepwise approach taken for our work. First, we constructed data sets for our analysis. Second, we classified and analyzed the contents of counterrumors. Finally, we verified the correlation between the number of counterrumors and the number of COVID-19 vaccine inoculators.

### Data Sets

#### Data of Counterrumors

We developed a system that collects and provides rumor information, called a “rumor cloud” [27], which indirectly collects rumor information through counterrumors. In our system, counterrumors include phrases such as “XXX is a rumor” and contain Japanese keywords such as “rumor” and “mistake.” We designate these keywords as “rumor markers.” Rumor markers used in this study were words that were listed in a Japanese thesaurus and were contained in rumor counters on Twitter after March 11 in Japan [27].

This system operates as follows: (1) the crawling function collects tweets that include a rumor marker from Twitter; (2) the judgment function extracts counterrumors from tweets collected in step 1. This function is constructed using a support vector machine and can judge counterrumors with approximately 85% accuracy. (3) The extraction function of rumor text extracts expressly provided rumor texts from counterrumors and stores them on our server. This function adopts pattern matching processing; (4) Rumor Cloud provides stored counterrumors and rumor texts.

The “rumor cloud” collects false rumors based on counterrumors; therefore, it is not possible to extract false rumors for which counterrumors have not been posted on Twitter. A previous study revealed a situation in which the existence of a rumor became widely known due to the number of posts that pointed out the rumor; interestingly, the rumor tweet itself received little traction or web-based engagement [28]. In other words, there are many people who respond to information they think is incorrect; additionally, counterrumors are considered to have more influence on users compared to a rumor that has not been indicated as a false rumor. Therefore, in this study, we used false rumors extracted through counterrumors as the targets of analysis.

**Figure 2:**
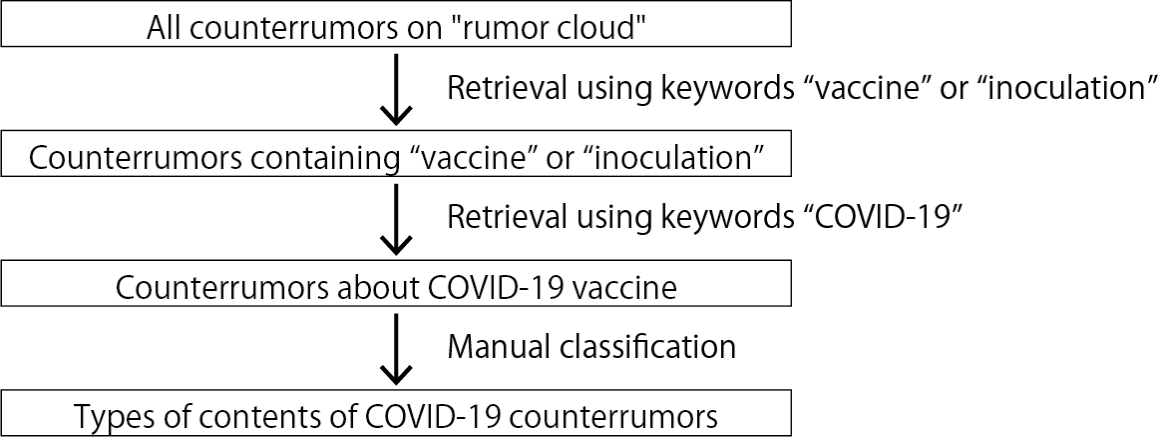
The procedure of our data extraction and classification.

Figure 2 shows the procedure for our data extraction and classification. We first extracted data that contained the keywords “vaccine” or “inoculation” in Japanese from the data set of counterrumors. There were days during the period when data could not be collected due to a problem with the server on which the system was installed; thus, these data were not included in the analysis. Next, we narrowed down counterrumors about the COVID-19 vaccine from extracted counterrumors that contain “vaccine” or “inoculation” using Japanese keywords that contain “COVID-19.” The number of counterrumors about the COVID-19 vaccine was 18,593 tweets.

#### Data for the Number of COVID-19 Vaccine Inoculators

We used statistical data on the number of vaccine inoculators published on the website of the Prime Minister’s office. Data for the number of vaccine inoculators by age group are updated once a week, and they contain only cumulative numbers. Therefore, we first collected the weekly updated data of vaccine inoculators by age group for each update from September 27, 2021, to August 15, 2022. Next, we constructed weekly vaccination data per age group by calculating the difference between the number of vaccinations in the previous week and the number of vaccinations in the current week. As a result of collecting and checking the publicly available data on the Prime Minister’s official website, the total number of vaccinations in the following week’s publicly available data was smaller than that in the previous week’s publicly available data, and the difference was negative in some cases. Since the cause of the decrease in the cumulative number of vaccinations is unknown, we treated the negative difference as a missing value. In this study, we used vaccination data for the following age groups: teenagers, 20-29 years, 30-39 years, 40-49 years, 50-59 years, 60-69 years, and 70 years or older.

### Classification of Contents of Counterrumors

#### Overview

We classify the contents of rumors in counterrumors. In this study, the term rumor is defined as information that is not well founded and whose authenticity is unknown or questionable. It is irrespective of the malicious intent that exists in the process of generation. Therefore, we regard information that contains phrases about indications of mistakes or questions by someone else as a rumor, and this study does not ask whether the information is really a rumor or not.

Table 1 shows examples of our data and classification results. Before the classification of counterrumors contents, we reviewed trends in the content of counterrumors. In this review, a reviewer checked all counterrumors by looking, and the target of classification is not counterrumors but the contents of rumors that are contained in counterrumors.

**Table 1:** Example of our data and classification results.

Counterrumor tweets	Contents of counterrumor	Sentiment polarities
There seems to be a false rumor spreading in some quarters that “life insurance will become void if people are vaccinated with the COVID-19 vaccine.” I wonder where the rumor that life insurance will become void if people are vaccinated with the COVID-19 vaccine is originated and on what basis.	Life insurance will become void if people are vaccinated with the COVID-19 vaccine.	Negative
To increase the vaccination rate, we should spread the false rumor that “COVID-19 vaccine makes hair thicker.?”	COVID-19 vaccine makes hair thicker.	Neutral

From this review, we found a tendency for counterrumors that they can be classified as including or not including contents about some influence or effect of vaccinations. Furthermore, the mentioned influence or effect could be classified in terms of sentiment polarities: negative, neutral, and positive. Therefore, we classify the contents of counterrumors through the following steps.

#### Step 1: Binary Classification (“Including” or “Not Including” Content About the Influence or Effect of the Vaccine)

In this step, we classified only the contents that indicate influences or effects of the COVID-19 vaccine directly as “including.” Even if they can be inferred as suggesting influences or effects, the contents without direct phrases were classified as “not including.” For example, consider the false rumor that “vaccines contain microchips.” Some people may believe that the vaccine will have an effect on their bodies if they are inoculated. However, there are no direct phrases about any influences or effects on people or society. Thus, this case was classified as “not included” in this study.

#### Step 2: Classification Based on Sentiment Polarities

In this step, we categorized the counterrumors classified as “including” in step 1 based on sentiment polarities. If the content of a counterrumor tweet contained clear expressions indicating “negative influences or effects on people or society” or “ineffectiveness,” it was classified as “negative.” If it contained clear expressions indicating “positive influences or effects on people or society” or “effectiveness,” it was classified as “positive.” If the contents are likely to be interpreted as good or bad by different people, they are classified as “neutral.”

### Verification of Correlation Between the Number of Counterrumors and COVID-19 Vaccine Inoculators

In some cases, vaccinations can be given without an appointment; however, in most cases, people are vaccinated only after taking an appointment. To consider this situation, the number of counterrumors may affect the number of vaccine inoculators later, rather than having an immediate impact on the day of the vaccination. In addition, vaccinated people themselves may post and spread counterrumors later. Therefore, in addition to correlation coefficients based on data from the same period, we also examined cross-correlation coefficients for different periods of counterrumors.

**Figure 3:**
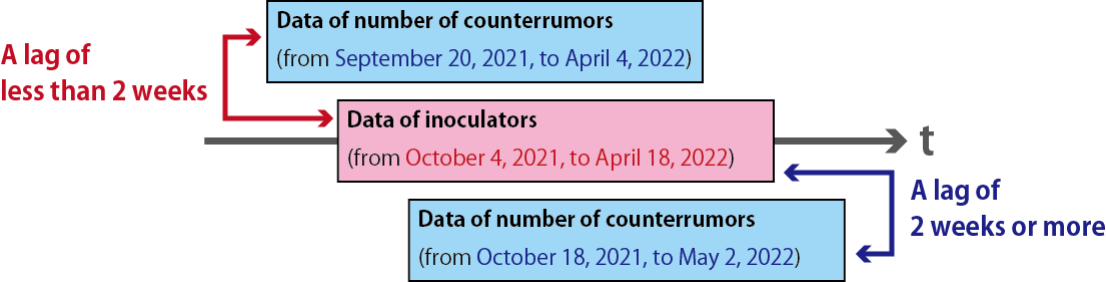
The example of setting lags between data of inoculators and data of number of counterrumors.

We verified the cross-correlation coefficient based on the following conditions:

1. The data from October 4, 2021, to April 18, 2022 (28 weeks), were used as the validation data with lag 0 (base period).
2. In the verification of correlation coefficients, we fixed the data period of inoculators to the base period. We set lags on the period of the data of the number of counterrumors. The lags were set per peak between 3 months before and 3 months after. Figure 3 shows an example of setting lags between data of inoculators and data of number of counterrumors.
  - In the case of a lag of approximately 2 weeks, we verified the correlation between the data of inoculators in the base period (data from October 4, 2021, to April 18, 2022) and the data of number of counterrumors shifted back by 2 weeks (data from September 20, 2021, to April 4, 2022).
  - In the case of a lag of more than 2 weeks, we verified the correlation between the data of inoculators in the base period (data from October 4, 2021, to April 18, 2022) and the data of number of counterrumors shifted by 2 weeks (data from October 18, 2021, to May 2, 2022).
3. We set the lag for verification between less than 12 weeks and more than 12 weeks.

### Ethical Considerations

In this study, we collected and used tweet data from Twitter’s application programming interface (API). Because the posts were made publicly, they were exempt from requiring institutional review board approval. Moreover, this study only included secondary data analysis of publicly available information and deidentified personal information. The Twitter API allows academic researchers with specific research objectives to obtain precise, complete, and unbiased data while protecting the security and privacy of Twitter users and the developer platform.

## Results

### Classification of Contents of Counterrumors

In this study, we classified the content of the counterrumors about vaccines into the following six types: (1) negative content indicating negative secondary influences or effects; (2) negative content indicating only “ineffectiveness;” (3) neutral content indicating some secondary influences or effects; (4) positive content indicating positive secondary influences or effects; (5) positive content indicating simply “effectiveness;” and (6) no mention of impact or effect.

First, 1 evaluator (evaluator A) classified all counterrumors into the 6 categories. Next, 2 different evaluators (evaluator B and evaluator C) performed the same classification task with 50 counterrumors in each category. Then, we checked the κ values between evaluator A and evaluator B and between evaluator A and evaluator C. Both κ values were higher than 0.7. Therefore, the results of the classification by evaluator A were used in this study.

Table 2 shows the classification results of counterrumors for the 6 categories and the examples of classified rumors. From Table 2, it can be observed that many counterrumors were classified as “negative content indicating negative secondary impacts or effects.”

**Table 2:** Classification results of contents of counterrumors.

Sentiment polarities and categories		Number of counterrumors (N=18,593), n	Example of classified rumors
**Negative**			
	Negative content indicating negative secondary influences or effects	15,078	Life insurance will become void if people are vaccinated with the COVID-19 vaccine.
	Negative content indicating only “ineffectiveness”	1171	COVID-19 vaccine is not effective against mutant strains.
**Neutral**			
	Neutral content indicating some secondary influences or effects	102	COVID-19 vaccine makes hair thicker. COVID-19 vaccine causes eyelids to project the latest animated images.
**Positive**			
	Positive content indicating positive secondary influences or effects	491	The COVID-19 vaccine can help you get back to your daily routine. As long as the COVID-19 vaccine is given, no one will get seriously ill.
	Positive content indicating simply “effectiveness”	496	COVID-19 vaccine will be effective for one year after vaccination. Apparently, an effective vaccine against COVID-19 is not BCGa but the pneumococcal vaccine.
**No evaluation**			
	No mention of influences or effects	1255	Vaccination for COVID-19 has not progressed. Those who participated in the election ballot were given is given priority for the COVID-19 vaccination.

^a^BCG: Bacille Calmette-Guerin.

### Verification Correlation Between the Number of Counterrumors and COVID-19 Vaccine Inoculators

Table 3 shows the results of the correlation coefficients between the number of counterrumors and COVID-19 vaccine inoculators. There was no strong correlation in the 70 years or older age group who did not take the Twitter usage rate surveys. By contrast, a significant and strong positive correlation was observed for the age groups: teenagers to 69 years of age at lags of −8 weeks, −7 weeks, and −1 week. This suggests that counterrumors posted and spread on Twitter can impact people’s vaccination behaviors.

**Table 3:** Cross-correlation coefficients between the number of counterrumors and the number of inoculators.

Lag (weeks)	Teenagers	20-29 years	30-39 years	40-49 years	50-59 years	60-69 years	70 years or older
−12	0.201	0.226	0.201	0.088	0.234	−0.044	0.275
−11	0.296	0.342	0.287	0.160	0.277	−0.014	0.266
−10	0.503	0.487	0.467	0.377	0.462	0.270	0.341
−9	0.710	0.668	0.665	0.617	0.656	0.559	0.507
−8	0.908	0.864	0.907	0.931	0.933	0.894	0.616
−7	0.906	0.848	0.884	0.906	0.911	0.880	0.608
−6	0.784	0.683	0.748	0.779	0.784	0.725	0.356
−5	0.451	0.542	0.540	0.498	0.577	0.495	0.517
−4	0.487	0.406	0.413	0.414	0.474	0.246	0.123
−3	0.511	0.461	0.478	0.451	0.504	0.419	0.274
−2	0.615	0.660	0.621	0.542	0.602	0.532	0.646
−1	0.802	0.725	0.761	0.773	0.782	0.718	0.404
0	0.641	0.611	0.644	0.624	0.650	0.586	0.430
1	0.645	0.617	0.641	0.631	0.651	0.588	0.478
2	0.576	0.564	0.608	0.619	0.643	0.587	0.414
3	0.215	0.023	0.038	0.163	0.049	−0.006	−0.076
4	0.056	0.046	0.055	0.180	0.068	0.236	0.036
5	0.085	0.147	0.123	0.254	0.100	0.188	0.140
6	0.073	0.055	0.070	0.213	0.073	0.132	−0.041
7	0.371	0.132	0.129	0.305	0.130	0.284	0.162
8	0.238	0.208	0.223	0.504	0.227	0.429	0.079
9	0.012	0.026	0.027	0.165	0.024	0.087	0.036
10	0.077	0.066	0.078	0.013	0.081	0.260	0.038
11	0.115	0.142	0.159	0.105	0.165	0.424	0.081
12	−0.059	−0.109	−0.106	−0.147	−0.101	−0.137	−0.110

## Discussion

### Principal Results

We found the following trends in the counterrumors about vaccines: (1) most counterrumors content about COVID-19 vaccines were negative, indicating negative secondary influences or effects. In the case of vaccines for emerging infectious diseases that people have not been vaccinated against in the past, negative contents may be easy to become a topic of counterrumors because people remain uncertain about the vaccine. (2) The cross-correlation coefficients between the number of counterrumors and vaccine inoculators showed strong positive correlations at lags of −8, −7, and −1 weeks. This suggests that the increase in the number of counterrumors may have been a factor in inducing inoculation behavior 1 week or 2 months later. Moreover, correlation coefficients of 0.5-0.6 were confirmed for lags of 1 week or more and 2 weeks or more. In other words, an increase in vaccine inoculators may have led to the posting or spreading of counterrumors 1 to 2 weeks later.

### Characteristics of Counterrumors’ Contents About COVID-19 Vaccines

Table 2 above shows that many counterrumors about vaccines contain contents that indicate negative secondary impacts or effects, such as “effects on the human body,” “effects on life and society,” and “nonserious.” Table 4 shows examples of topics mentioned in the specific contents of many counterrumors. “Specific contents” in Table 4 were classification results that a reviewer checked and classified all counterrumors by looking at. People, especially those who have not been vaccinated in the past, still remain uncertain about the COVID-19 vaccine. Therefore, false rumors mentioning the “negative secondary impacts or effects” rather than the “no effect” of the vaccine were spread; many counterrumors were also posted on Twitter.

**Table 4:** Examples of topics that mentioned in specific contents of many counterrumors.

Specific contents	Example of topics with many counterrumors
Contents related to effects on the human body	“Infertility,” “Emission of toxic substances from the body,” “Elimination of the effects of other vaccines,” “Death,” and “Gene alteration.”
Content related to impact on life and society	“I won't be able to get an MRI^a^” or “my life insurance will be invalidated.”
Nonserious content	“Becoming connected to 5G” and “a magnet will stick to the vaccinated arm.”

^a^MRI: magnetic resonance imaging.

### Relation Between Number of Counterrumors and Vaccine Inoculators

We discuss the results suggested by the strong correlations identified in this study separately for negative and positive lags. A negative lag indicates that the number of past counterrumors affects later inoculation. Our results suggest that an increase in the total number of counterrumors leads to an increase in the number of vaccinations about 1 week or 2 months later. As mentioned above, many counterrumors about the COVID-19 vaccine contain negative contents. Thus, the increase in the number of counterrumors about the negative contents of the vaccine may have contributed to the inducement of vaccination behavior by changing the attitudes of those who viewed the counterrumors.

A positive lag indicates that the number of past inoculators affects the later increase or decrease of the number of counterrumors. In this study, significant correlation coefficients of 0.5 to 0.6 were found for all age groups, from teenagers to the 60-69 years age group, for lags of 1 week or more and 2 weeks or more. These results suggest that the number of counterrumors increases as the number of vaccinations increases. In other words, people may post or spread counterrumors on Twitter after being vaccinated and not experiencing the adverse effects described in the rumors.

### Comparison to Previous Work

COVID-19 is an emerging infectious disease that has spread in an unprecedented manner after the widespread use of social media platforms. As previously stated, the results of a survey on vaccine hesitancy suggest that the use of social media and the internet are primary sources of information and are factors associated with vaccine hesitancy [18]. Browsing through information on social media has been shown to lead to vaccine hesitancy in Canada [19]. However, previous studies had not discussed the specific effects of social media on vaccinations using quantitative data. Our findings reveal the specific relations between social media and vaccinations. We think that these would be a foundation for considering countermeasures of vaccination.

### Limitations

First, the results obtained in this study were only based on the analysis of counterrumors. However, a greater variety of information may be posted on social media, and information not included in this analysis may have different characteristics and influences.

Second, other media outlets, such as the various news websites on the internet, television, newspapers, and oral reports, can also influence people’s vaccination intentions. It is difficult to analyze and verify the influence of these media outlets separately, and even more difficult to verify whether the increase in counterrumors, which is the subject of this study, had an effect on people’s vaccination behavior. However, a statistically significant positive and strong correlation was confirmed, and we believe it is possible to consider our results a factor that can be used to promote vaccination.

### Conclusions

We analyzed counterrumors about the COVID-19 vaccination posted on Twitter, a medium prone to the posting and spreading of unverified information. COVID-19 is an emerging infectious disease that has spread in an unprecedented manner, and vaccination is key to controlling the outbreak. To promote vaccination, it is important to analyze the factors, such as social media, that may influence people’s vaccination behavior and consider how to respond to them. We found that most counterrumors about COVID-19 vaccines were negative, indicating negative secondary influences or effects. Moreover, the increase in the number of counterrumors may have been a factor in inducing inoculation behavior 1 week or 2 months later, and an increase in vaccine inoculators may have led to the posting or spreading of counterrumors 1-2 weeks later.

We think that these results reveal that social media is one of the elements that can be used to promote vaccination. Moreover, we believe that our findings would be a foundation for considering countermeasures of vaccination. In the future, it will be necessary to conduct analyses using larger and more diverse data sets and to analyze characteristics not revealed in the present data.

## Abbreviations

API: application programming interface
SAGE: Strategic Advisory Group of Experts
WHO: World Health Organization

## Data Availability

The data used in this study are available by collecting tweet data from Twitter’s API and the publicly available data on the Prime Minister’s official website [29].
